# Enhanced fresh and hardened properties of foamed concrete modified with nano-silica

**DOI:** 10.1016/j.heliyon.2024.e25858

**Published:** 2024-02-12

**Authors:** Md Azree Othuman Mydin, P. Jagadesh, Alireza Bahrami, Anmar Dulaimi, Yasin Onuralp Özkılıç, Roshartini Omar

**Affiliations:** a School of Housing, Building and Planning, Universiti Sains Malaysia, 11800, Penang, Malaysia; b Department of Civil Engineering, Coimbatore Institute of Technology, Tamil Nadu, 638 056, India; c Department of Building Engineering, Energy Systems and Sustainability Science, Faculty of Engineering and Sustainable Development, University of Gävle, 801 76 Gävle, Sweden; d Department of Civil Engineering, College of Engineering, University of Kerbala, Karbala, 56001, Iraq; e Department of Civil Engineering, College of Engineering, University of Warith Al-Anbiyaa, Karbala, 56001, Iraq; f Department of Civil Engineering, Faculty of Engineering, Necmettin Erbakan University, 42100, Konya, Turkey; g Department of Civil Engineering, Lebanese American University, Byblos, Lebanon; h Department of Construction Management, Faculty of Technology Management and Business, Universiti Tun Hussein Onn Malaysia, Parit Raja, Batu Pahat, Johor, 86400, Malaysia

**Keywords:** Foamed concrete, Nano-silica, Pozzolanic compositions, Intrinsic air permeability, Compressive strength, SEM analysis, Chloride penetration

## Abstract

Nowadays, the application of nanotechnology has gained increased attention in the concrete technology field. Several applications of concrete require light weight; one such concrete used is foamed concrete (FC), which has more voids in the microstructure. In this study, nano-silica (NS) was utilized, which exhibits a pozzolanic nature, and it reacts with other pozzolanic compositions (like lime, alumina, etc.) to form hydrated compounds in concrete. Apart from these hydrated compounds, NS acts as a filler material and enhances properties of concrete such as the fresh and hardened properties. This research examines the fresh, hardened, and microstructural properties of FC blended with NS. The ratio of binder and filler used in this research is 1:1.5, with a water-to-binder ratio of 0.45 and a density of 880 kg/m^3^. A total of six different weight fractions of NS were added to FC mixes, namely 0%, 1%, 2%, 3%, 4%, and 5%. Properties assessed for FC blended with NS were the slump, bulk density, strength parameters (flexural, splitting tensile, and compressive strengths), morphological analysis, water absorption, and porosity. It was concluded from this study that the optimum NS utilized to improve the properties was 3%. Apart from this, the relationship between the mechanical properties and NS dosages was developed. The correlations between the compressive strength and other properties were analyzed, and relationships were developed based on the best statistical approach. This study helps academicians, researchers, and industrialists enhance the properties of FC blended with NS and their relationships to predict concrete properties from other properties.

## 1 Introduction

There is a need to change the practice of concrete production and use to ensure that concrete production stays eco-friendly in the future [1]. As an alternative to the current convention of utilizing natural aggregates and cement, which are major sources of carbon dioxide and other greenhouse gas emissions worldwide, it is recommended to use mineral admixtures, supplementary cementitious materials, and other sustainable materials [[2], [3], [4], [5]]. As a result, excessive consumption of natural resources would be reduced in the long run. For concrete production, however, to maintain concrete quality, the requirement of mineral admixture is promoted nowadays [6,7]. Compared to conventional construction, there are a lot of advantages, like cost savings, reduction in self-weight, excellent fire resistance, etc., associated with the usage of foamed concrete (FC), which pushes to use it in the global building sector [[8], [9], [10], [11], [12], [13], [14]]. Typically, FC is referred to as a porous or cellular lightweight material that is suitable for an extensive variety of applications in construction [15,16].

There are various densities and compressive strengths of FC, most commonly ranging from 450 kg/m^3^ to 1950 kg/m^3^, as well as different densities and compressive strengths [[17], [18], [19], [20], [21], [22]]. It is noteworthy, however, that while FC is widely accepted, it does have some downsides, such as high permeability, high shrinkage, brittleness, and a higher probability of cracking because of its permeable structure, primarily associated with voids, which restrict its application to building construction in terms of load-bearing requirements [23,24]. FC has been found to be volatile in many studies, especially when its density declines [25]. Even small variations in the FC density can have a substantial influence on the FC's durability and performance. A decrease in the FC density resulted in an increase in cavities with larger dimensions [26]. Furthermore, FC is prone to breaking at low densities due to weak bonds and more voids [27]. To make the foam more stable, silica fume is added at the liquid foaming stage in FC [28]. The strength of FC was modeled with the help of various mechanical properties [29,30]. Maglad et al. [31] found that the distribution of the pore size of FC is different, influencing the properties (mechanical and durability) of FC. Smaller sizes of pores result in a strength increase for FC, and the effect of different variables on the density of FC is reported by Nambiar et al. [32].

Since FC comprises a greater number of closed cells with voids, it is problematic to enhance both the strength and durability properties related to water characteristics by maintaining their quality. This problem can be addressed by adding mineral admixtures or supplementary cementitious materials. One such material recommended by the researchers is silica fume, which speeds up the cement hydration and optimizes the pore structure for FC [33]. Nano-silica (NS) is an inorganic chemical substance with a particle size to the ultrafine of about less than 20 nm, which has various tremendous beneficial properties like the nucleus effect in the crystals, pozzolan effect, and morphological action, and it is helpful in improving the concrete performance [28]. Nanoparticles that are spread out tend to create crystallization environments that are good for concrete hydrate crystallization. This makes sure that the hydration reactions happen quickly. It is important to note that they are ideal as fillers as they occupy voids within the concrete particles, which aids in lowering the porosity of the material [34]. Nanoparticles that are evenly and well-spaced are said to help smaller crystals grow. This causes the microstructure to be denser, which makes it less likely that cracks and interlocking will happen. As a result, the material's ability to harden is improved, resulting in better performance as a hardened material.

The mechanical and durability properties of concrete are enhanced by adding NS, as reported by researchers [[35], [36], [37]]. In order to reduce the consumption of cement for the production of high-strength concrete [38], this also helps preserve the environment from pollution and reduce costs [39]. A complete review of the usage of NS in concrete to improve the properties of concrete and its advantages was presented by Jagadesh et al. [40]. Role of processing technique in enhancing the amount of silicon dioxide in the pozzolanic material improves the properties of cementitious composites [41,42]. Enhanced fresh properties are due to the high surface area of NS particles, and part of the surface area in NS starts hydrating, resulting in an increase in the fluidity of the mix [43]. The increase in the compressive strength of concrete with the incorporation of NS is owing to the dense microstructure. A stronger interfacial transition zone (ITZ) because of the addition of NS contributes to the increase in the splitting tensile strength and flexural strength of concrete [43].

The increase in need for water, as the percentage of NS in concrete increases, is due to smaller particle sizes, which leads to a higher surface area and instantaneous interaction between NS and liquid phase of the cementitious matrix system [44,45]. From this point, it can be concluded that with an increase in NS, there is a decrease in the workability of concrete. The increase in the compressive strength with the addition of NS is owing to the calcium silicate hydrate (C-S-H) formation as a result of the pozzolanic reaction between calcium hydroxide and silicon dioxide in NS. This also results in an increase in the density and a reduction in the porosity [46]. An increase in the splitting tensile strength with an increase in NS is due to the void reduction in the cementitious matrix, and the formation of a strong ITZ leads to a denser microstructure [47]. Proper distribution of NS in the cementitious matrix enhances the flexural strength because of filler characteristics and dense ITZ between the cement paste and aggregate [48]. The improved modulus of elasticity of concrete with the addition of NS is owing to the proper distribution of NS particles in the cementitious paste and the larger surface area of smaller NS particles [49]. The addition of NS results in the formation of fewer pores in the C-S-H gel, leading to a denser microstructure that contributes to the enhanced mechanical and durability properties of concrete.

Different sizes of nanomaterials were used for the performance enhancement of concrete under various conditions confirmed in the literature [50,51]. During the cement hydration, NS combines with lime to develop the C-S-H gel, which improves the hardness and elasticity of concrete, thus increasing its mechanical strength. The hydration process is fastened, and a denser microstructure can be created, enhancing the strength properties of the cement paste if NS is well mixed with concrete. With more energy on their surface, nanoparticles may agglomerate in large quantities, resulting in non-uniform particle dispersion. The filler effect of nano- and micro-scaled silica particles fills the pores between the pore grains and contributes to the concrete's properties. Hou et al. [52] experimented the pozzolanic properties of NS by examining the kinetics and morphology of the reaction. Because of the compact gel structure that NS causes, the cement's rate of hydration slows down as it ages. A cement matrix should contain between 1% and 5% of NS to prevent any agglomeration of NS. The concrete's mechanical properties were improved by using NS up to 3%, as stated by Zhuang and Chen [53]. The NS addition increases the compactness of the concrete hydration products and enhances the compressive strength and resistance against frost [54]. The addition of NS from 3% to 15% to FC enhances the compressive strength and durability of concrete [55]. Nanoceramics, though, were found to improve the concrete's mechanical properties by up to 6%, according to Mas et al. [56]. Nano-metakaolin's recommended proportion for use in concrete varies from 6% to 10% for cement paste, 3%–10% for cement mortar, and 10% for conventional concrete [57].

### 1.1 Research significance

There has been little research conducted so far to estimate the influence of NS on the durability and strength properties of FC. Although there are limited numbers of commercially available NS for use in FC, they are among the most promising NS for this type of application. There have been some studies on the addition of NS to concrete, but there are still several uncertainties regarding the mechanism by which NS might be able to change the characteristics of concrete. The ambiguity in this situation needs to be addressed. Hence, it is essential to examine the effects of NS modification on the fresh concrete and hardened concrete properties of FC. Confirmation of the contribution of the enhanced mechanical properties is proven by the morphology analysis using a scanning electron microscope (SEM).

### 1.2 Research objectives

This investigation intends to respond to this need by doing a planned inquiry to find a solution for the effect of NS on FC. It is intended to determine the strength and durability properties of FC influenced by the NS addition. The enhancement of FC properties is confirmed by the SEM study. It is necessary to develop an association between the dosage of NS and the mechanical properties of FC. Studies are further extended to the relationship between the mechanical properties of FC. Relationships are required because they have many advantages, like a reduction in cost, time savings, etc.

## 2 Materials and methods

### 2.1 Materials

**Fig. 1:**
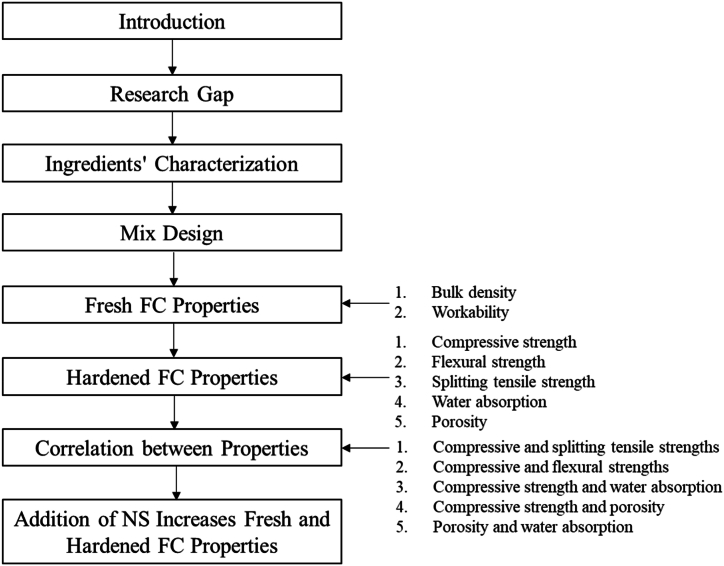
Methodology of study.

There were five main ingredients required to produce the FC specimens, which were ordinary Portland cement (OPC) as a binder, fine aggregate as a filler, clean water, and a protein surfactant. The methodology of the present study is depicted in Fig. 1.

#### 2.1.1 OPC

The FC mixes were enriched with NS as an additive to enhance their performance. According to BS-EN 197–1 [58], OPC CEM1 is suitable for a broad range of applications. The specific gravity of used CEM1 is 3.16, and the fineness of cement as per the Blaine fineness value of 380 kg/m^2^ is found as per specifications of BS 12 [59]. The compressive strength of CEM1 on the 7^th^ and 28^th^ days of testing was experimentally obtained as 34.5 MPa and 45.2 MPa, respectively. Table 1 summarizes the chemical compositions of OPC employed in this study.

**Table 1:** Chemical compositions of OPC.

Components	Percentage
Calcium oxide (CaO)	59.95
Silicon dioxide (SiO_2_)	21.33
Aluminum oxide (Al_2_O_3_)	5.79
Magnesium oxide (MgO)	3.59
Sulfur oxide (SO_3_)	2.95
Iron oxide (Fe_2_O_3_)	2.91
Potassium oxide (K_2_O)	0.5
Sodium oxide (Na_2_O)	0.19
LOI	2.79

#### 2.1.2 Fine aggregate

**Fig. 2:**
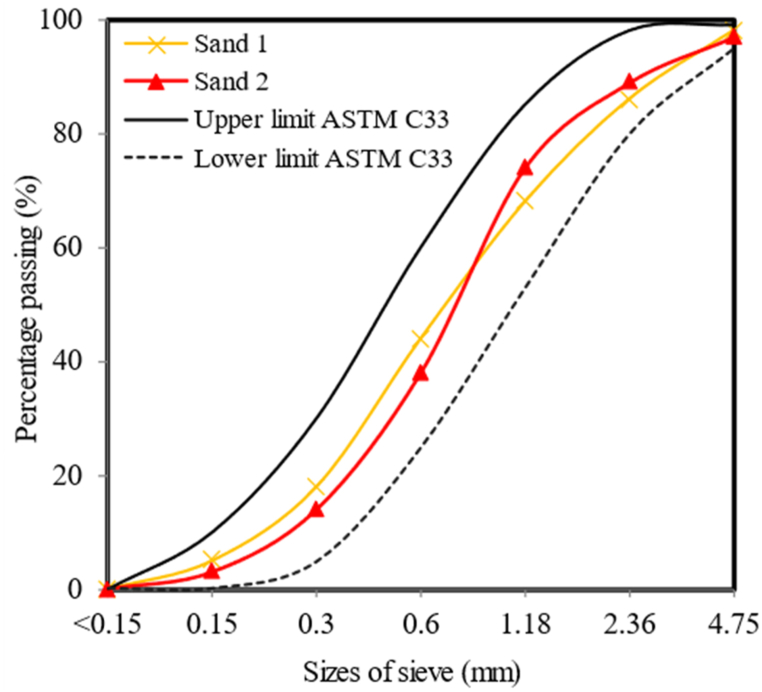
Sand grading curve of fine aggregate.

In addition to the quality of the used sand, the composition of the mortar mix can also influence the properties of the mortar slurry. Fine sand was utilized as a filler in FC mixes. ASTM C33-03 [60] was employed to determine the particle size distribution. Fig. 2 shows the sand grading curve of fine aggregate used in this study. A maximum grain size of 5 mm is found in 2.71% of the product. In terms of the uniformity, it has a value of 2.69 and a curvature coefficient of 1.08. For mixing purposes, it was necessary to use tap water that followed the conditions of BS-EN 3148 [61]. Dried sand was sieved through a 2.36-mm sieve as per BS 882 [62]. The estimated specific gravity of sand was found to be 2.65 [63]. The water absorption of sand was achieved as 1.25% [63]. The moisture content of sand was resulted as 3% [64].

#### 2.1.3 Water

Water that was used for mixing the components was unadulterated and free of any filth or other forms of organic matter. For this investigation, a water-cement ratio of 0.45 was selected since achieving acceptable FC workability was possible with this ratio in earlier experiments [65].

#### 2.1.4 NS

**Fig. 3:**
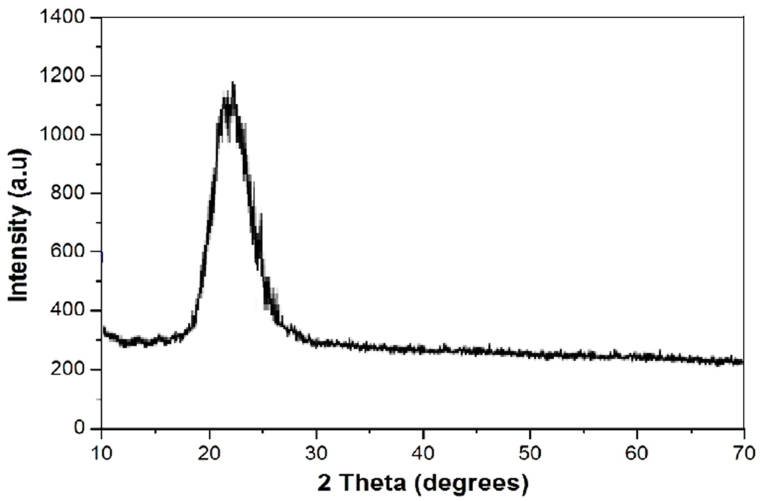
XRD pattern of NS.

NS was acquired from Nano Life Quest Sdn. Bhd., Malaysia. Fig. 3 illustrates the x-ray diffraction (XRD) spectra of NS. In the XRD test of the NS sample, a clear and broad peak was seen at a 2-theta angle of 23.5°. This finding suggests that the particles of NS exhibited an amorphous morphology. Table 2 presents the physical properties of NS.

**Table 2:** Physical properties of NS.

Properties	Value
Specific gravity	2.15
Density (kg/m^3^)	1970
Fineness modulus	2.19
Moisture absorption (%)	0.39
Bulk porosity (%)	23.5

#### 2.1.5 Foaming agent

There is an adjustable foam nozzle on the foam generator, which can discharge a required amount of foam at a set rate ranging from 4 to 12 cubic feet per minute, depending on the level of flow. It is recommended that the foam density be between 75 kg/m^3^ and 85 kg/m^3^. A mortar slurry mix can be prepared as soon as the density has been confirmed to be within the acceptable range. Protein foaming agents were used to reduce the apparent friction of a solution and increase the firmness of the foam by reducing the apparent friction of the solution. There was a ratio of 1:35 between the protein surfactant and clean water utilized in the application of the foam. It was necessary to dilute 1 L of protein foaming agent with 35 L of water to produce a steady foam. To generate the foam, a foam generator was employed.

### 2.2 Mix proportions

FC with a density of 880 kg/m^3^ was prepared in the experiments. As displayed in Table 3, FC contains various weight fractions of NS, corresponding to the mixture proportion of FC. It was determined that the cement-sand ratio would remain at 1:1.5, and the water-cement ratio would remain at 0.45. There were six FC mixes in total that were produced, specifically NS0 (control), NS1, NS2, NS3, NS4, and NS5, which respectively represent varying weight fractions of FC additions as 0%–5% in the final mix.

**Table 3:** FC mix proportions containing varying proportions of NS.

Mix	NS (%)	NS (kg/m^3^)	Sand (kg/m^3^)	Cement (kg/m^3^)	Water (kg/m^3^)	Foam (kg/m^3^)
NS0	0	0.0	497.0	331.3	149.1	34.3
NS1	1	10.1	497.0	331.3	149.1	34.3
NS2	2	20.2	497.0	331.3	149.1	34.3
NS3	3	30.4	497.0	331.3	149.1	34.3
NS4	4	40.5	497.0	331.3	149.1	34.3
NS5	5	50.6	497.0	331.3	149.1	34.3

**Fig. 4:**
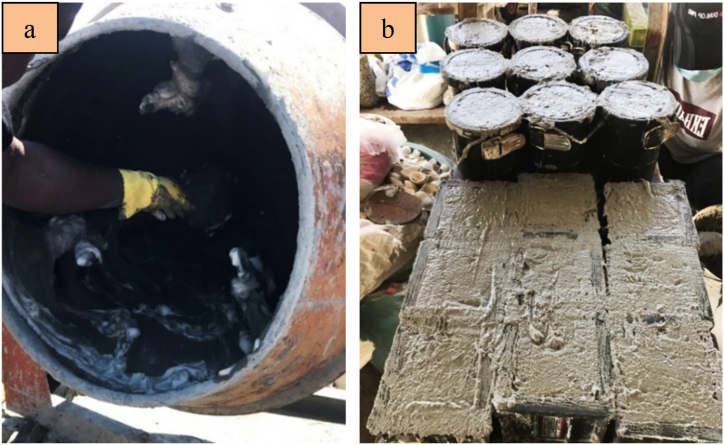
Preparation of FC mix.

**Fig. 5:**
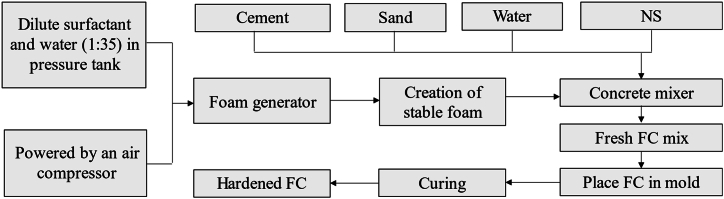
Flow chart for preparation of FC mix.

Fig. 4a indicates the preparation of the FC mix. As soon as the fresh FC mix was blended homogeneously, it was poured into steel molds, as demonstrated in Fig. 4b. The flow chart for preparation of the FC mix is depicted in Fig. 5.

### 2.3 Methods

**Fig. 6:**
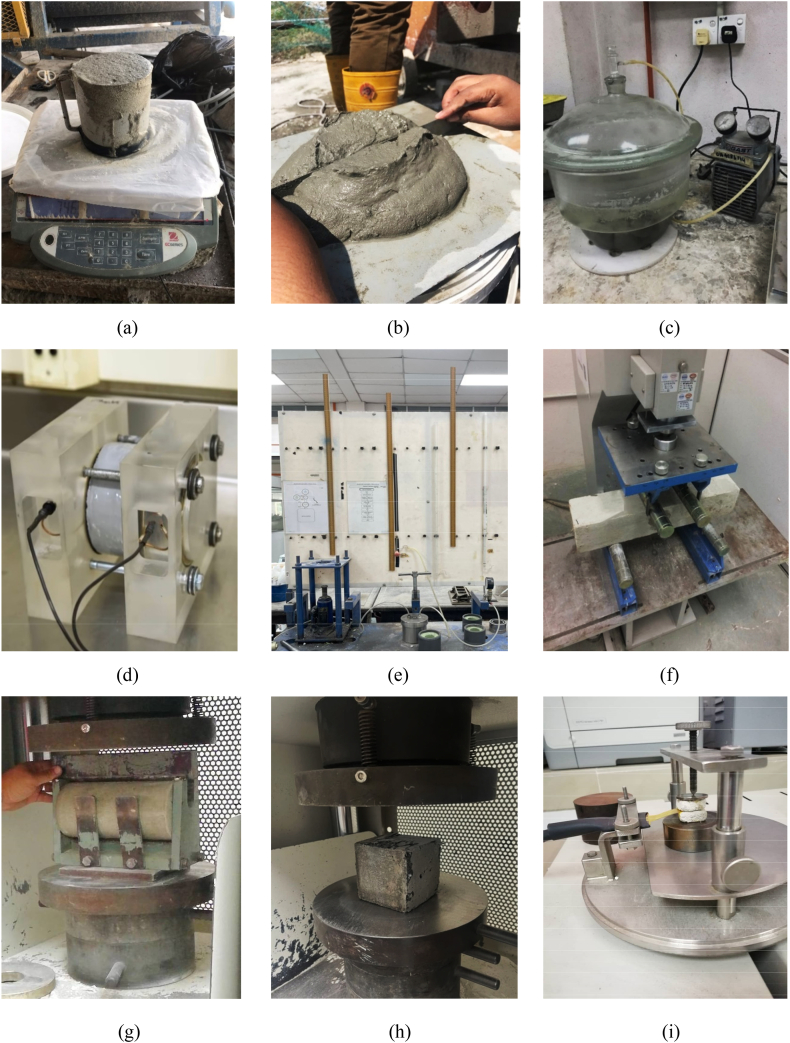
Tests conducted in this study.

This section provides an overview of the testing procedures utilized to evaluate the properties of FC with varying concentrations of NS. The workability, density, water absorption, porosity, intrinsic air permeability, chloride diffusion, flexural strength, compressive strength, thermal conductivity, thermal diffusivity, and specific heat capacity are some of the properties that were investigated. In accordance with the specifications outlined in BS EN 12350-6 [66], varying densities of the FC mixture were observed at different stages of the mixing procedure (Fig. 4). In line with ASTM C230-97 [67], a flow table test was performed to assess the flowability of FC mixtures, as shown in Fig. 6a and b. In order to verify the porosity of FC, a vacuum saturation approach was employed [68], as can be seen in Fig. 6c. Additionally, a water absorption test, complying with BS EN 1881-122 [69], was conducted to meet the requirements set forth. Following that, a chloride diffusion test (Fig. 6d) was done to see how well FC resisted the entry of chloride ions, following the steps mentioned in ASTM C1202 [70].

After following the steps suggested by Mas et al. [56], the test of intrinsic air permeability was carried out, as illustrated in Fig. 6e. The flexural strength test utilized 100 × 100 × 500 mm FC prisms in accordance with the specifications of BS EN 12390-5 [71]. The experimental arrangement for the four-point bending test is displayed in Fig. 6f. Then, the splitting tensile test was performed following the specified techniques delineated in BS EN 12390-6 [72]. Cylindrical specimens with a diameter of 100 mm and a length of 200 mm were used for the purpose of conducting tests, as presented in Fig. 6g. The compression test (Fig. 6h) was done employing FC cubes measuring 100 × 100 × 100 mm, as specified by BS EN 12390-3 [73].

A hot plate with directed heat conduction was also used to test the FC samples' thermal properties, such as their thermal conductivity, specific heat capacity, and thermal diffusivity. The utilized methodology aligns with the guidelines outlined in ASTM C177-19 [74]. The experimental arrangement for the thermal properties test is illustrated in Fig. 6i.

## 3 Results and discussion

### 3.1 Bulk density

**Fig. 7:**
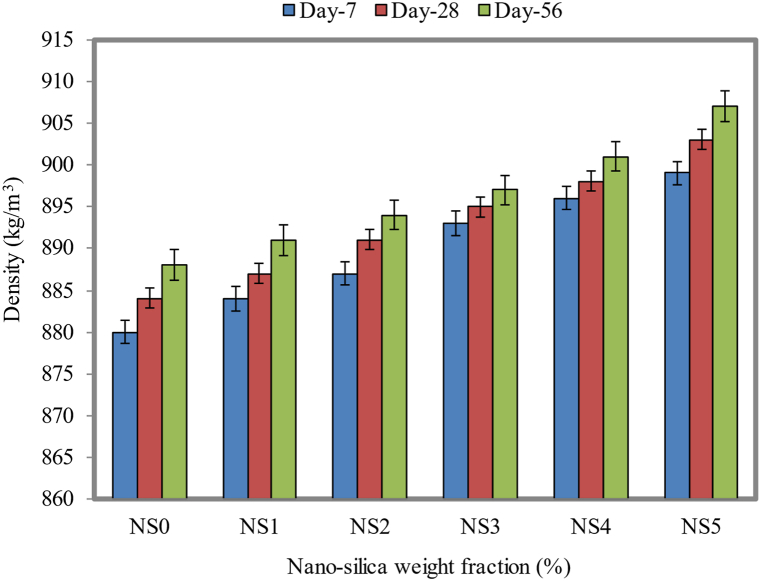
Bulk density of FC at different weight fractions of NS.

The FC density increased slightly from day 7 to day 56 on account of the increase in the NS weight fraction from 1% to 5%, as pointed out in Fig. 7. In contrast to the control mix, the FC mix comprising a 5% weight fraction of NS had a density of 903 kg/m^3^ on day 28, while the control FC mix had a density of 884 kg/m^3^. On day 7 of curing, FC with 2% NS had a density of 887 kg/m^3^, while it had a density of 891 kg/m^3^ on day 28 and a density of 894 kg/m^3^ on day 56. Despite this issue, the density was still satisfactory on day 56 of the experiment. There were differences in the density between NS-blended FC (NS1, NS2, NS3, NS4, and NS5) and control specimens (NS0) on day 28 as 3, 7, 11, 14, and 19 kg/m^3^respectively.

In the FC cementitious matrix, NS within it has a higher specific gravity, which is responsible for the increased density. Further, it is also possible to improve grain-filling density with the addition of NS to FC, reduce the proportion of solid fragments that remain void, and increase the amount of free water that can be used for lubrication with NS [46]. The opposing processes may result in an increase in the viscosity. The porous nature of pozzolanic material decreases the density of concrete, as reported in the literature [75]. When NS is added to FC, it often causes an upsurge in the amount of water used, as well as a rise in the stress and viscosity [76]. As a filler for the FC matrix, NS reduces the porosity and cavities, therefore, FC can become denser.

### 3.2 Workability

**Fig. 8:**
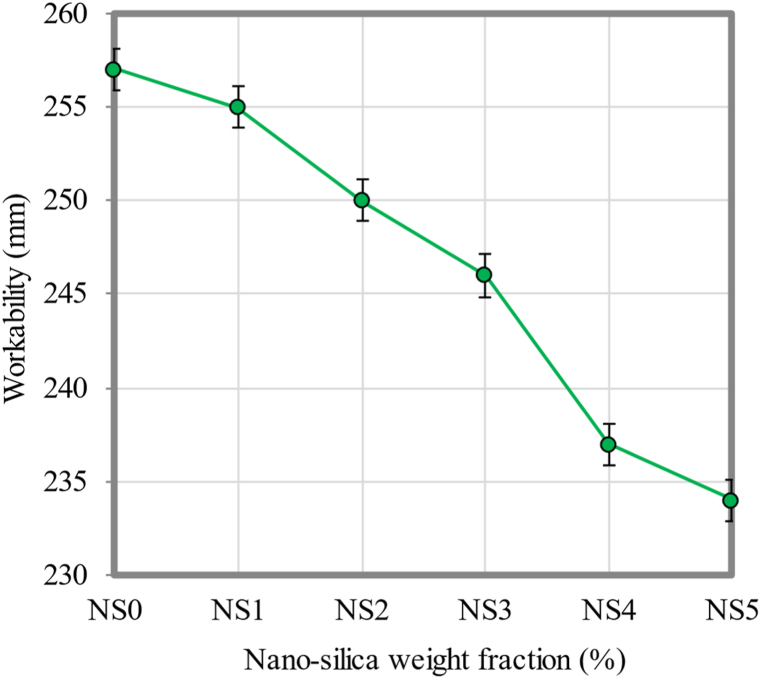
Workability of FC at different weight fractions of NS.

In fresh conditions, a high-quality FC should have a workability of approximately 220–250 mm. A slump is required to develop sufficient strength. Overall, the bigger the determined slump flow, the better the workability of FC is going to be as well. In general, this indicates that FC flows easily, but at the same time, it is not segregated in any way. Because FC is capable of self-compacting, it is vital that FC has a satisfactory degree of the workability to achieve its maximum strength. The slump flow of FC mixes decreased as the NS content increased, as demonstrated in Fig. 8. 1% NS FC had the largest slump flow diameter of 255 mm, whereas the smallest slump flow diameter of 234 mm was logged for FC with the presence of a 5% NS, which recorded the smallest slump flow. Contrary to the control FC mix, which had a slump flow of 257 mm, FC containing NS had a decreased slump flow. There have been several studies where NS was observed to have a similar effect on the workability [77,78].

As NS weight fractions increase, fluidity decreases. There is an obvious variation in the workability of FC containing 1% NS compared to FC that was used as a control. An increase in NS, resulting in an increase in water demand in the FC mix, was observed. This adjustment has been made for the primary reason that the NS medium slurry disperses uniformly and may be a noticeable factor in improving the particle size dispersion since it is faster to spread [79]. The addition of NS encourages the cement hydration in addition to speeding up the setting of cement. NS increases the water demand in FC as the proportion of NS increases. Due to their high fineness and low specific surface area, the NS grains would be able to take in a significant amount of water. This would allow them to have a greater capacity for absorption. In addition, since the NS grains react with the liquid-face cement matrix so quickly, this would enhance their ability to absorb water [80].

### 3.3 Water absorption

**Fig. 9:**
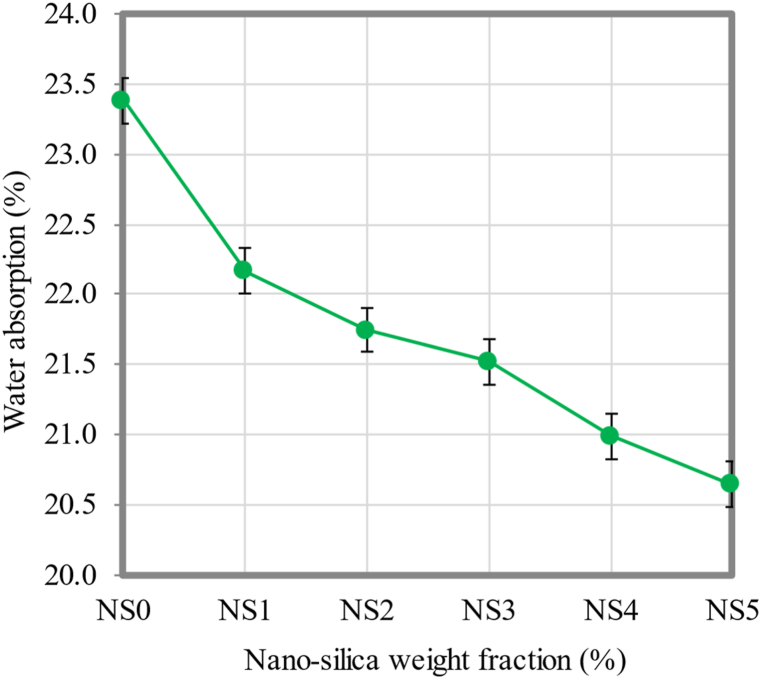
Water absorption of FC at different weight fractions of NS.

The water absorption behavior of FC was one of the factors employed to establish the FC porosity. The water permeability of FC was determined by this criterion. The total water absorption rate of FC, to some extent, is a function of its porosity and compactness. In FC, high water absorption rates can have a negative effect on the durability of the harmful ions and solutes that have penetrated through the pores and penetrated the interior of the cementitious matrix along with water. Fig. 9 depicts the water absorption test results of FCwith varying NS weight fractions. With reference to Fig. 9, it was found that the water absorption rate was decreased when NS was included in this investigation. The FC specimen with 5% NS (NS5) had the lowest water absorption rate value, which was decreased by 11.7% in comparison to the control specimen (NS0). NS declined the water absorption of FC based on the obtained results, since it had a better microstructure, as noted in the morphological analysis. Due to the smaller particle size of NS in the FC mix, a seal was formed between the gel pores within the mixture.

**Fig. 10:**
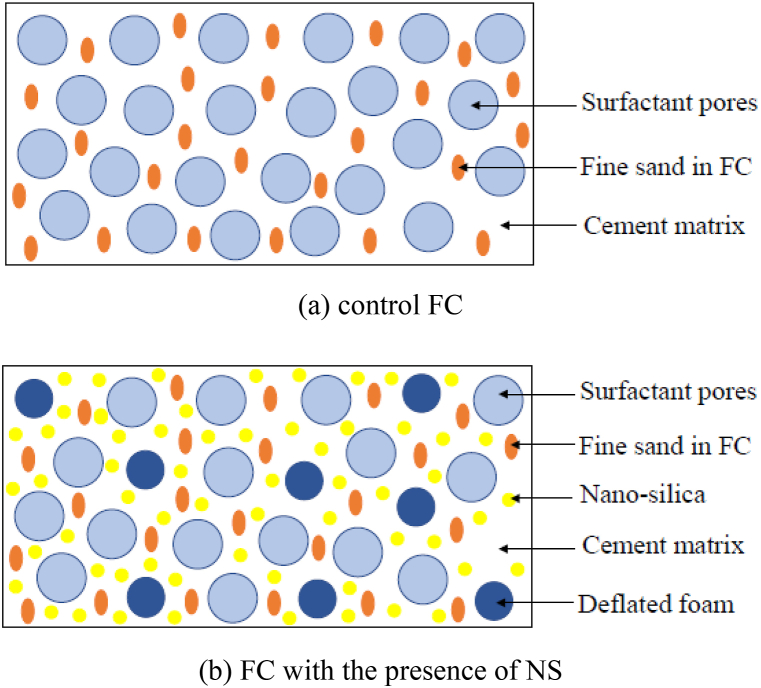
Schematic diagram of FC-NS mixes.

This point was due to the refined pores as well as the densified microstructure of the surface [80]. Deflating the size and quantity of pores through the presence of NS improved the microstructure of FC. On top of that, it made the bond between the cement matrix and sand stronger, which made FC stronger (Fig. 10b) compared to the control mix (Fig. 10a). Additionally, the presence of NS in FC resulted in the creation of filled pore arrays, which caused the diameter of spaces to be reduced, which is displayed in a schematic diagram (Fig. 10).

### 3.4 Porosity

**Fig. 11:**
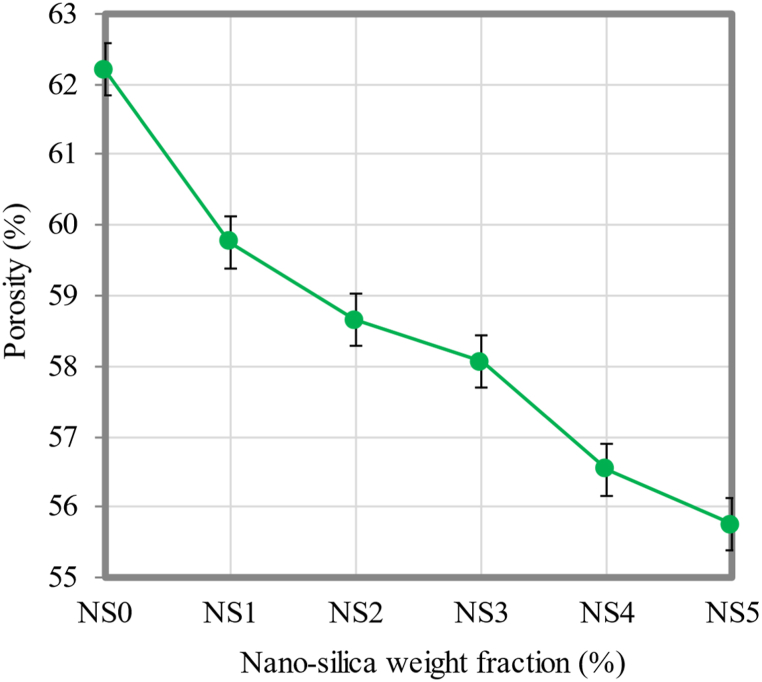
Porosity of FC at different weight fractions of NS.

**Fig. 12:**
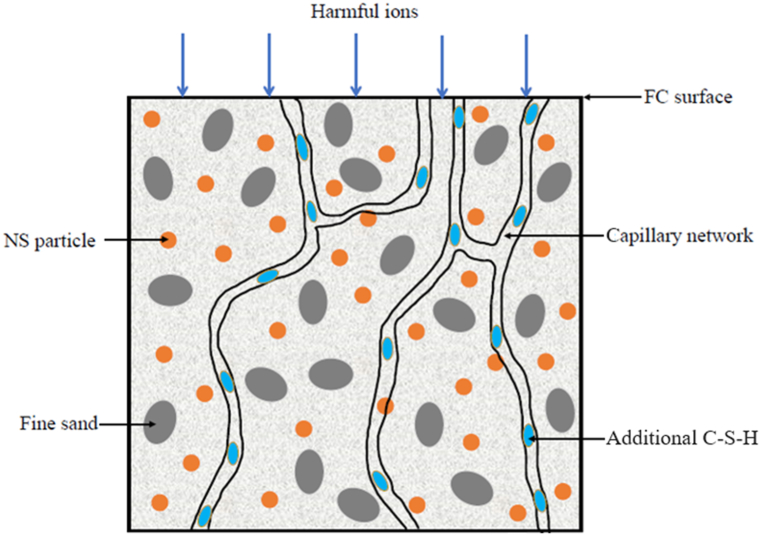
Mechanism of NS enhancement in FC.

The ability to characterize the characteristics of water transport by the permeable medium through capillary networks depends on the porosity of FC. Fig. 11 shows the results of measurements used to determine the porosity of FC containing varying NS weight fractions. As the weight fractions of NS were greater in the FC mixtures, the porosity was considerably reduced. As compared to the control FC mix, the porosity was improved by 10.4%, 9.1%, 6.7%, 5.7%, and 3.4%, respectively, with the addition of 5%, 4%, 3%, 2%, and 1% of NS associated with the control FC mix. Since the pores between the binder paste and the filler interface were filled with NS, capillary pore activity was reduced. It may be clarified that the pores were packed with NS. The reason for such a trend in porosity values is also owing to a modification of the microstructure in the presence of NS, as discussed earlier. NS is utilized to fill the discrete and continuous voids present in the specimens. This lowers the capillary suction that draws water into the FC specimens. The transition zone between fine sand and binder contains numerous gel pores [81]. When water invaded these pores, water and air were likely to form at the interface. Cement-based material is well known for its ability to migrate water through capillaries, which leads to the capillary action. Due to the interface and the capillary action, the formation of the pores is different in each region. The addition of NS reduces these pore formations. Moreover, the presence of NS promoted the mortar's microstructure densification and reduced its porosity, particularly the connectivity of pores within the matrix [82]. As illustrated in Fig. 12, incorporating NS refines the pore structure, thereby improving the impermeability of FC. There are numerous capillary networks found within FC that facilitate the diffusion of dangerous ions into the matrix.

When evenly spread-out units of NS nanoparticles are added, they can act as crystallization and nucleation points for hydration reactions. This makes the crystallization and growth of hydration products better. As a result of the reactivity of NS, plenty of additional C-S-H and other gels were generated. Due to these gels, the pores were refined, and a discontinuous pore structure was developed by interconnecting hydration products. The calcium hydroxide (C-H) crystals were restricted in size during this process because of the large amounts of C-H consumed. Due to the microaggregate effect, the NS and C-S-H particles also develop a denser bond between aggregates and binder in the cementitious matrix. The pore size was improved, and the pore structure became more inhomogeneous and complex. Consequently, FC was more resistant to deterioration owing to its denser microstructure and discontinuous pore structure, making it more difficult for harmful ions to infiltrate.

### 3.5 Chloride diffusion

**Fig. 13:**
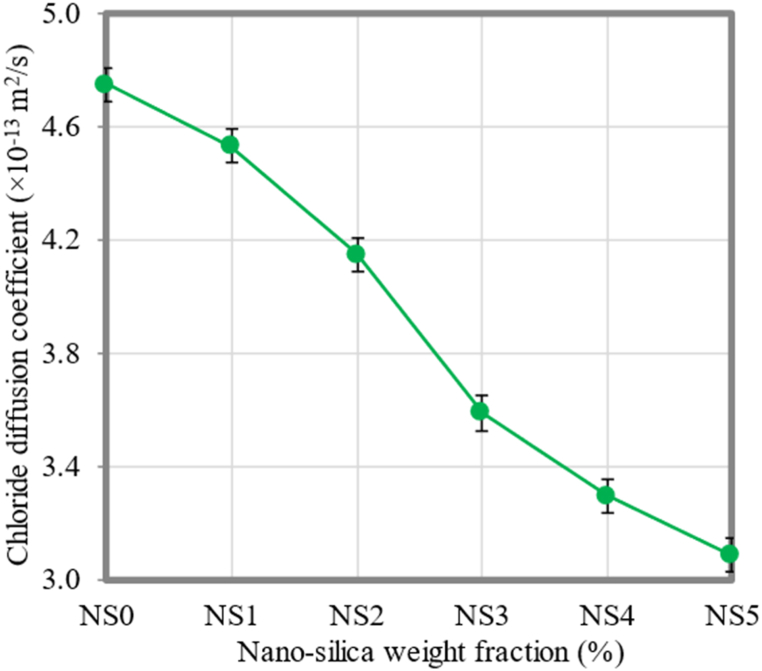
Chloride diffusion coefficient of FC at different weight fractions of NS.

Fig. 13 depicts the chloride diffusion coefficient of FC with different quantities of NS incorporated. It was found what the chloride diffusion coefficient was for FC mixtures with 1%, 2%, 3%, 4%, and 5% NS, and that coefficient was compared to the control mixture. The use of NS in FC was found to improve its resistance against the chloride ion penetration. The FC mixtures containing NS exhibited enhanced values of the chloride diffusion coefficient when compared to the control FC mixture. The chloride diffusion coefficient of the control FC mixture was quantified and recorded at 4.75 × 10^–13^ m^2^/s. The chloride diffusion coefficient indicated reductions of 4.63%, 12.63%, 24.42%, 30.53%, and 34.95% when incorporating different percentages of NS (1%, 2%, 3%, 4%, and 5%), respectively. Previous studies have reported that the addition of NS to cement-based materials leads to improvements in various key characteristics. These improvements encompass a decrease in the porosity, an increase in the tortuosity, and a greater amount of solidified C–S–H gel [83]. The enhancements have been demonstrated to be crucial in reducing the rate at which chlorides infiltrate the FC material. The densification of the microstructure of FC (as referenced in Section 3.11) occurs as cement hydrates and advances.

### 3.6 Intrinsic air permeability

**Fig. 14:**
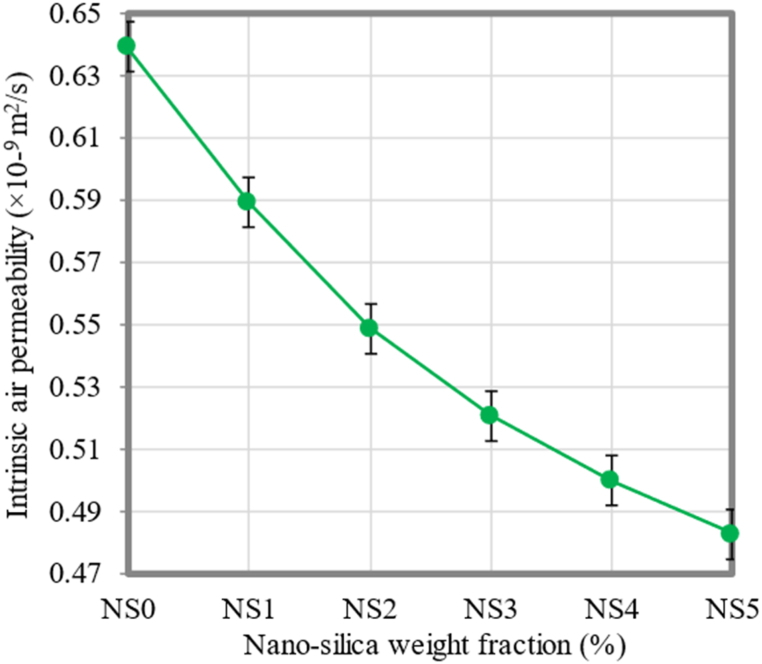
Intrinsic air permeability of FC at different weight fractions of NS.

Fig. 14 shows the intrinsic air permeability of FC containing different percentages of NS. The intrinsic air permeability of FC mixtures containing 1%, 2%, 3%, 4%, and 5% NS was determined and contrasted to that of the control mixture. When compared to the control FC mixture, all FC mixtures incorporated with NS exhibited lower intrinsic air permeability values. The intrinsic air permeability for the control FC mixture has been recorded as 0.639 × 10^−9^ m^2^/s. Adding 1%, 2%, 3%, 4%, and 5% of NS, the intrinsic air permeability was reduced by 7.82%, 14.08%, 18.47%, 21.76%, and 24.41%, respectively. This implies that NS functions as an accelerator for the process of the cement hydration while also serving as a substance that enhances the internal microstructure of FC.

Consequently, the microstructure of FC exhibited improved uniformity and reduced porosity due to the incorporation of NS [84]. When the FC mixture consists of a relatively high proportion of NS, the adhesion properties of the mixture are enhanced, while the internal porosity of FC is reduced [85]. Consequently, there is a reduction in the gas permeability. The enhanced filler effect of NS can be attributed to the incorporation of nano-sized fine particles. Additionally, the high pozzolanic activity of NS significantly augments the formation of the C-S-H gel. It is possible that improving the microstructure in ITZs could change the composition of the C-S-H gel, which would make it much less permeable to air. These reactions lead to an increase in the homogeneity and strength of ITZs, as well as a decrease in their diameter. This results in a reduced probability of microcracks and a more uniform distribution of particles, which ultimately contributes to the refinement of the grain structure in the hydrated cement paste within ITZs [86]. Therefore, the incorporation of NS into cement has the potential to lead to the reorganization and enhancement of the physical and chemical microstructures of FC.

### 3.7 Flexural strength

**Fig. 15:**
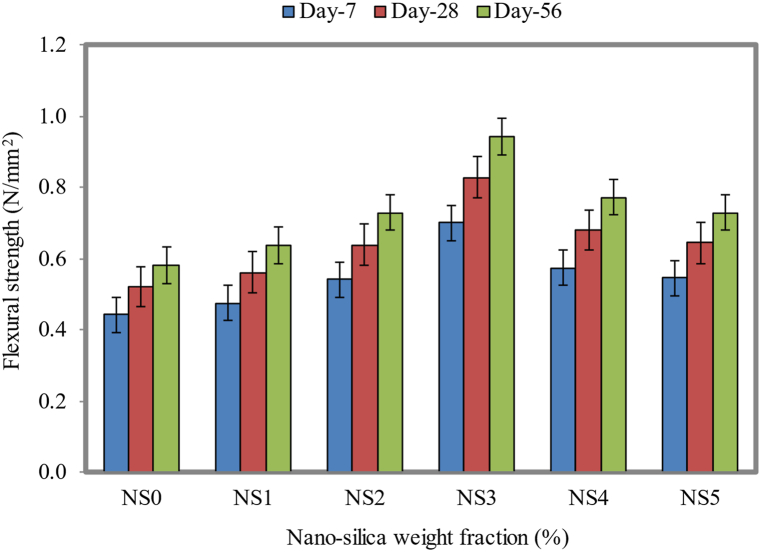
Flexural strength of FC at different weight fractions of NS.

Based on Fig. 15, it is noted that the flexural strength was 0.44, 0.47, 0.54, 0.70, 0.57, and 0.55 MPa at 7 days of testing. When the curing period was increased to 28 days, the flexural strength was improved to 0.52, 0.56, 0.64, 0.83, 0.68, and 0.64 MPa for the dosages of NS as 0%, 1%, 2%, 3%, 4%, and 5% in FC, respectively. For further increasing the curing period to 56 days, the flexural strength was increased to 0.58, 0.64, 0.73, 0.94, 0.77, and 0.73 MPa for the NS dosages of 0%, 1%, 2%, 3%, 4%, and 5% in FC, respectively. Approximately 59%, 60%, and 62% enhancements of the flexural strength were achieved for the NS3 mix compared to the NS0 mix, as obtained for 7, 28, and 56 days, respectively.

Due to their superlattice structure and high van der Waals forces at the interface, NS shows a strong bond with the cement matrix. NS also makes a dense structure of hydration products, which improves the distribution of particle sizes during hydration and decreases the size and connectivity of pores, which enhances the bending strength during hydration [87]. Particles that are not spread out evenly may also clump together and agglomerate, which can lead to lower the bending strength after adding 3% NS to FC. To strengthen the bond between the fillers and binders at ITZ, adding NS to the matrix during the manufacturing process can increase the packing density of FC cementitious matrices [88]. Owing to their high specific surface area and fine grain size, NS incorporated in cement-based materials may accelerate the C-S-H gel formation. In comparison to the control FC mix, all the NS-modified FC specimens exhibited the improved bending strength.

### 3.8 Splitting tensile strength

**Fig. 16:**
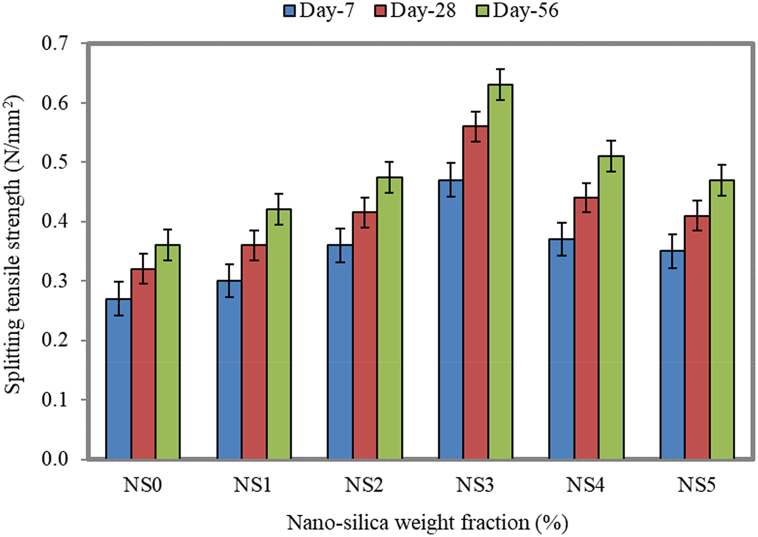
Splitting tensile strength of FC at different weight fractions of NS.

For the dosages of NS as 1%, 2%, 3%, 4%, and 5%, there was an increase in the splitting tensile strength as 12.5%, 29.8%, 75.0%, 37.5%, and 28.1%, respectively, compared to the control specimen (NS0) for 28 days of curing, whereas for 56 days, there was an enhancement in the splitting tensile strength as 16.7%, 31.8%, 75.0%, 41.7%, and 30.6%, respectively, compared to the control specimen, as illustrated in Fig. 16. The presence of NS in the cement matrix causes the formation of H_2_SiO_2_, which later reacts with the available calcium ions to form additional C-S-H, with the resulting C-S-H products being dispersed in water and space between C-S-H being filled by NS, leading to the formation of denser C-S-H products [89]. The increase in the splitting tensile strength for a similar dosage of NS was reported by Fallah et al. [90]. The FC specimen with 3% of NS had the splitting tensile strength of 0.47, 0.56, and 0.63 MPa, which displays the highest strength for 7, 28, and 56 days. An increase in the strength is found to enhance the interface between the concrete matrix and aggregates [91]. The size of NS plays a key role in the increase in the strength because the particles then distribute randomly across the surface, which is available for the filling and further reaction processes. The influence of several parameters on the increase in the splitting tensile strength was presented by Martinez-Garcia et al. [92].

### 3.9 Compressive strength

**Fig. 17:**
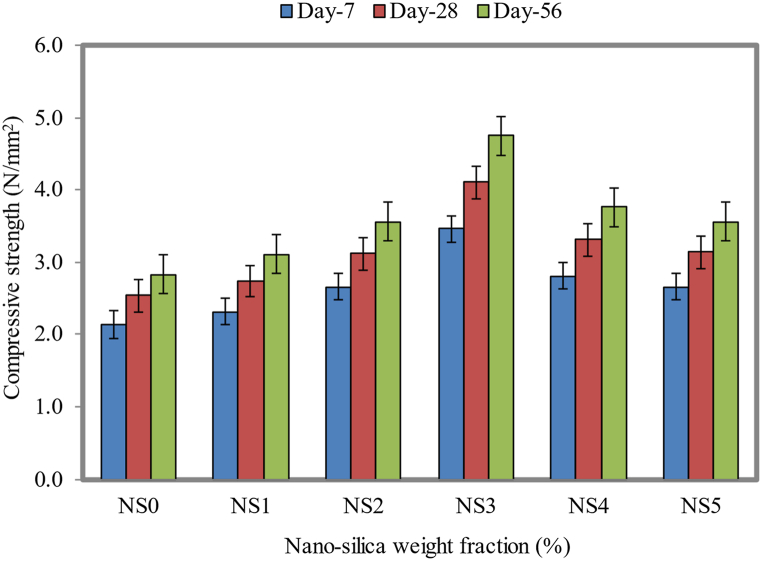
Compressive strength of FC at different weight fractions of NS.

Fig. 17 indicates that adding 3% of NS to FC resulted in the optimal compressive strength. A 56-day compressive strength of 4.75 N/mm^2^ was attained by adding 3% NS to the material, as compared to the control specimen at 2.83 N/mm^2^, which represents a 67.8% improvement. Results from this study are consistent with those concluded by Amin and Abu El-Hassan [93]. The compressive strength of FC was increased from 7 to 56 days when 3% NS was added to the cement weight. As related to the NS0 mix, the compressive strength was enhanced by about 37%.

The increase in the compressive strength may have been caused by NS elements reacting with C-H fragments in lime to make an extra layer of the C-S-H gel. In the FC cementitious matrix, these elements work synergistically to increase the compressive strength. In addition, nanoparticles with a large specific area are susceptible to secondary interactions with the cluster [94]. Nevertheless, a concrete mix without NS will not be capable of hydration to a trace quantity of C-S-H when it is hydrated by cement. C-S-H plays a crucial role in the strength development. Consequently, cementitious materials that do not contain NS exhibit low compressive strength due to their lack of NS. After 7, 28, and 56 days of curing, FC reached the maximum strengths of 3.46, 4.11, and 4.75 N/mm^2^, respectively. A superficial reaction between the C-H compounds and NS generates additional C-S-H gel.

For the dosages of NS as 1%, 2%, 3%, 4%, and 5%, there was an increase in the compressive strength of about 7.9%, 22.8%, 61.8%, 30.3%, and 23.6% for 28 days of curing period, respectively, compared to the control mix, whereas for 56 days, there was an increase in the compressive strength of about 10.2%, 25.8%, 67.8%, 32.9%, and 25.8%, respectively. There is a large amount of complexity associated with NS because of its nanoscopic scale, huge interfacial area, and arrangement of atoms. These atoms can migrate rapidly under external stress, absorbing energy during the entire process, and restraining deformation during this process [95]. There are many microcracks that form within FC when force is applied to it, leading to FC fractures that are characterized by small and major cracks that occurred because of the applied force. If internal fractures appear, grow, and coalesce, the FC matrix loses its load-bearing surface. In contrast to this, NS improves the fracture resistance and crack propagation of the FC specimens.

### 3.10 Thermal properties

**Fig. 18:**
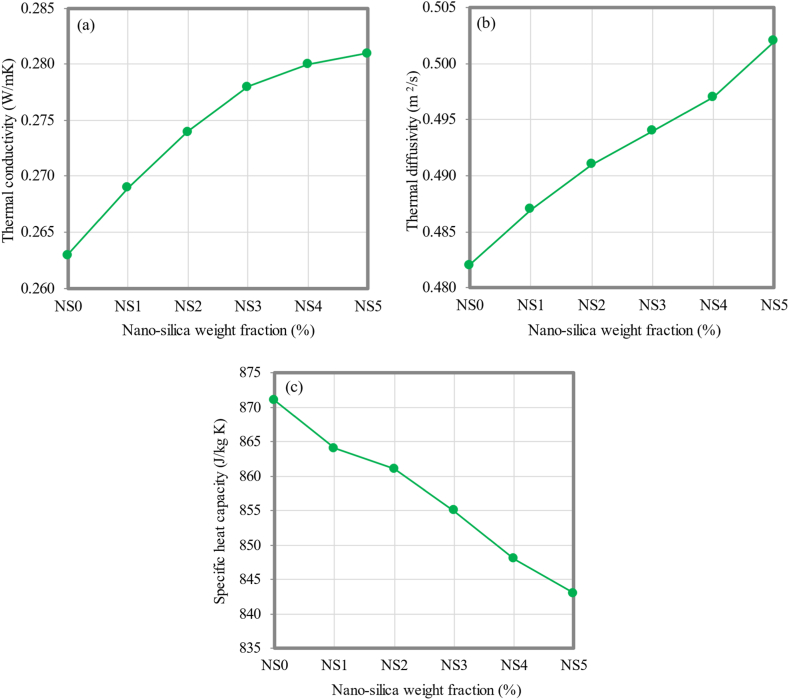
Thermal properties of FC at different weight fractions of NS; (a) thermal conductivity, (b) thermal diffusivity, (c) specific heat capacity.

Fig. 18(a–c) depicts the thermal properties parameters of FC with different proportions of NS. The thermal conductivity, thermal diffusivity, and specific heat capacity of FC mixtures with varying percentages of NS (1%, 2%, 3%, 4%, and 5%) were measured and compared to those of the control FC mixture. Overall, the incorporation of NS into FC has been found to enhance its thermal properties. The thermal conductivity values of the FC mixtures with NS were found to be higher when compared to the control FC mixture. The thermal conductivity of the control FC mixture has been quantified and recorded as 0.263 W/mK. When different amounts of NS (1%, 2%, 3%, 4%, and 5%) were added, the thermal conductivity was increased by about 2.28%, 4.18%, 5.70%, 6.46%, and 6.84%, respectively. Furthermore, the thermal diffusivity of FC showed a similar pattern to that of the thermal conductivity. The thermal diffusivity demonstrates an upward trend with the rise in the NS fractions inside FC. The thermal diffusivity of the control FC mixture has been measured to be 0.482 m^2^/s.

**Fig. 19:**
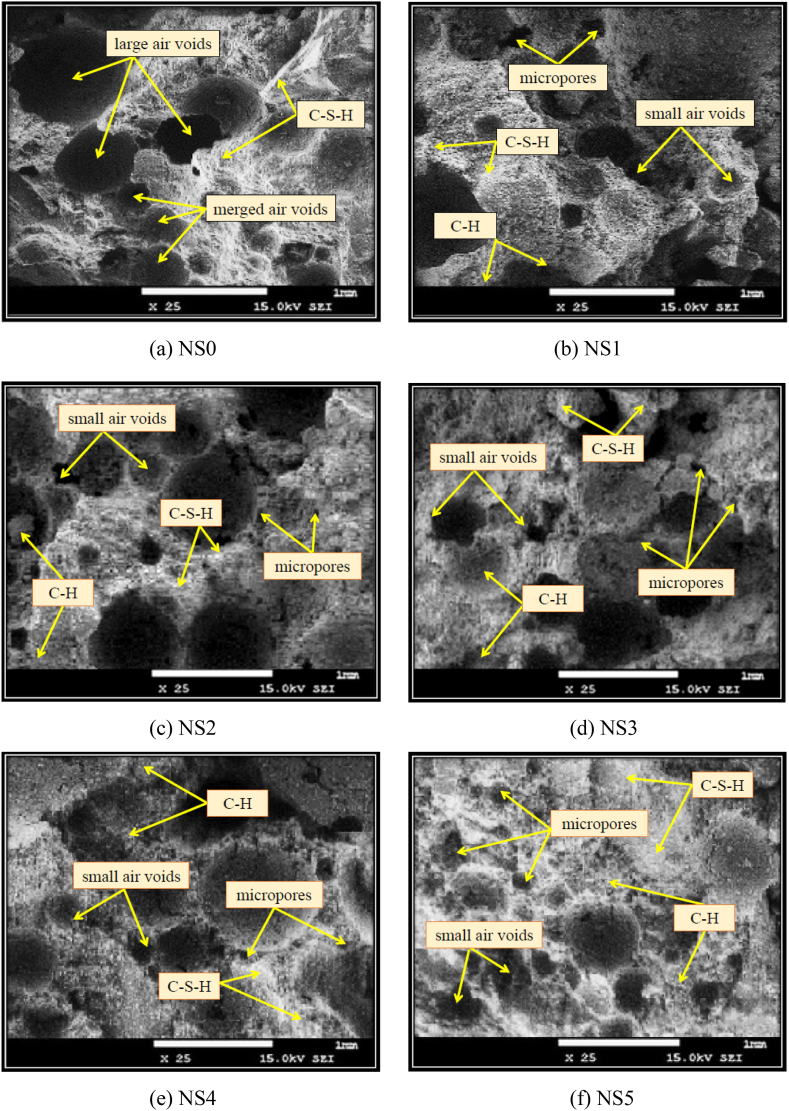
SEM micrographs of FC containing varying weight fractions of NS.

The thermal diffusivity exhibited improvements of 1.04%, 1.87%, 2.49%, 3.11%, and 4.15% after integrating 1%, 2%, 3%, 4%, and 5% of NS, respectively. On the other hand, it was observed that the specific heat capacity of FC displayed a decrease as the proportions of NS were increased. The specific heat capacity value of 871 J/kg K was measured for the control FC mix. The specific heat capacity of FC dropped to 864 J/kg K, 861 J/kg K, 855 J/kg K, 848 J/kg K, and 843 J/kg K, with the corresponding presence of 1%, 2%, 3%, 4%, and 5% of NS. The incorporation of NS into FC led to a notable rise in the thermal conductivity, suggesting an improvement in the alteration of the pore structure. The observed phenomena can be attributed to the reduced pore size in the FC specimens containing NS in comparison to the control FC specimen. As a result, there was an increase in the insulation of the heat conduction. Microstructure analysis has observed the phenomenon of the comparability, as stated in Section 3.11. The control specimen indicates the presence of merging air bubbles, as illustrated in Fig. 19a. This phenomenon has the potential to decrease the heat conductivity because of the low thermal conductivity exhibited by air [96].

### 3.11 Morphology analysis

Fig. 19 depicts SEM images of FC encompassing differing weight fractions of NS. According to the SEM micrograph of the control FC (NS0), hydrated products are bound and connected to the C-S-H gel. Large air pores and merged air voids can be clearly seen (Fig. 19a). Furthermore, the microstructure of FC containing NS1 to NS5 showed that compact and dense hydration products were produced, and there was a decrease in the C-H crystals (Fig. 19a–f). During the hydration process, it appears that there is a chemical reaction between NS and the free lime.

Since the particles of NS are so fine in comparison to the C-S-H gel, this results in a reduction in the size of air voids, as noted in Fig. 19b and (c). NS grains have a diameter of around 50 nm, which makes them suitable for reacting as a nucleus, since their size is conducive to this. This will lead to thick and hydrated products being created with an increased ITZ. A C-S-H gel is formed by the pozzolanic reactivity of NS with lime and reduces the pores, thereby contributing to an increase in the density and mechanical properties, as elucidated in Fig. 19d. A particle of NS is approximately 120 times finer than a grain of cement, and it has the ability to fill gaps in the cement matrix, resulting in a denser matrix [97]. There is greater homogeneity and density in the microstructure of cementitious materials with NS when compared to cementitious materials without NS [98]. NS can be used to demonstrate improved microstructural properties in concrete due to its effects on the interface, filler, and filler interactions.

In addition to enhancing the hydration processes of concrete, NS can also serve as an activator, forming large volumes of the C-S-H gel by activating the process of hydration. Thus, NS is a valuable substance for enhancing the durability of cement paste by lowering calcium leaching and reducing calcium leaching. The NS nucleation action improved the FC porous form. Incorporating NS into the hardened paste resulted in a significant modification of the hydration behavior of the paste as well as changes in its microstructure. From Fig. 19e and (f), it is seen that there is a formation of pores with a higher diameter compared to that of a 3% dosage of NS.

### 3.12 Correlation between mechanical properties

A comparison of the predicted values (from the literature) and the experimental values is required nowadays to assess the best equations suitable to predict the mechanical properties. It is necessary to check the proposed equation with the experimental values from the literature, which confirm the proposed equations with the help of sensitive assessment. Sensitive assessment is the technique used to compare the predicted values to the experimental values. The assessment technique utilized in this study is the coefficient of determination (R^2^) to evaluate the quality of the proposed equation. Considering the cost, time-consuming nature, saving natural resources, preventing manpower, and complexity of testing, many researchers, building codes, and standards are interested in predicting unknown properties. The equations are proposed in such a manner that the simplified method has accuracy.

#### 3.12.1 Correlation between compressive strength and splitting tensile strength

**Fig. 20:**
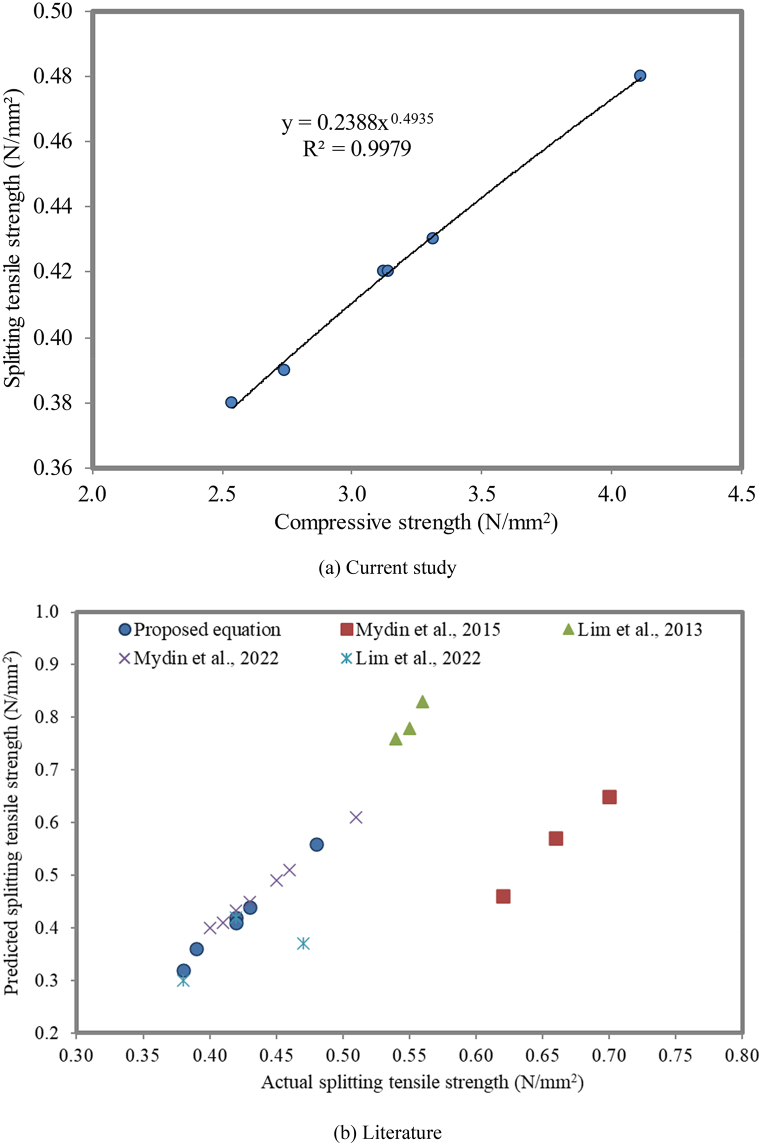
Relationship between compressive strength and splitting tensile strength.

For conventional concrete, the relationship between the compressive strength and splitting tensile strength is developed using the power type equation proposed in the literature [99]. The relationship between the splitting tensile strength and compressive strength of conventional concrete and self-compacting concrete was similar [100]. The correlation between the compressive strength and splitting tensile strength of geopolymer concrete with the help of a confidence interval was analyzed [42,101]. Tensile stress separations in the specimen lead to the loss of the contact between aggregate grains at the failure section. In the case of FC, if there is an absence of fine aggregate, the aggregate interlock cannot function properly [102]. Fig. 20 (a) displays a strong linear correlation (R^2^ = 0.9979) between the splitting tensile strength and compressive strength of FC on the 28^th^ day of the curing period. This infers that as the compressive strength of FC increases, its splitting tensile strength also increases. The coefficient of determination (R^2^) value is found to be 0.996 between the splitting tensile strength and compressive strength of FC blended with NS, which is proposed in equation (1). The error value is defined as the difference between the actual splitting tensile strength and the predicted splitting tensile strength. To obtain the best equation, the error value should be kept near zero.

(1) $$Splitting\;tensile\;strength=0.238\sqrt{Compressive\;strength}$$

In order to verify the linear relationship between the splitting tensile strength and compressive strength of FC blended with NS, the compressive strength and splitting tensile strength from the literature [[103], [104], [105], [106]] were collected. The splitting tensile strength values obtained from the literature were incorporated into the linear equation (equation (1)) to estimate the corresponding compressive strength values of FC.

These predicted splitting tensile strength values were compared to the experimentally determined values, as illustrated in Fig. 20b. This comparison provides valuable insights into the accuracy of the proposed linear relationship between the splitting tensile strength and compressive strength. It is found that a linear relationship exists between the actual (experimental) and predicted compressive strengths based on the strength values observed. From this point, it can be concluded that the equation proposed is satisfying the results from the literature. The summation of the error value for Mydin et al. [103] shows the lowest error value, which indicates that the predicted splitting tensile strength is the best. In addition to Mydin et al. [105], the experimental values from Lim et al. [106] present the best relationship.

#### 3.12.2 Correlation between compressive strength and flexural strength

**Fig. 21:**
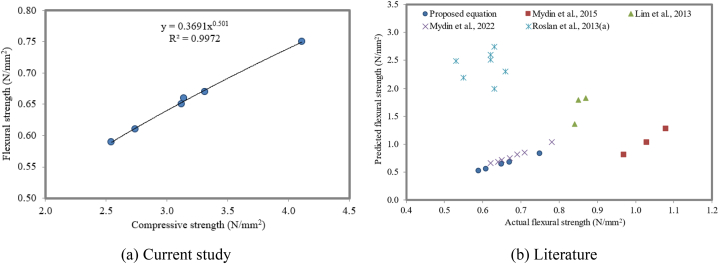
Relationship between compressive strength and flexural strength.

The relationship between the compressive strength and flexural strength displays a direct relationship between them. Predicted flexural strength is calculated as a product of the constant and square root of the compressive strength. The correlation between the compressive strength and residual flexural strength of steel fiber-reinforced concrete was proposed by Ruiz et al. [107]. Verification of the relationship between the compressive strength and flexural strength of concrete with the help of palm kernel shell concrete and geopolymer concrete was confirmed by Yusuf et al. [108], Vanathi et al. [42], and Jagadesh et al. [101]. An increase in the compressive strength results in an increase in the flexural strength. It is also reported that the increase in the strength is due to the increase in the density at ITZ with an increase in NS, which will contribute to an increase in the aggregate paste bond. Fig. 21a illustrates a strong positive linear correlation between the compressive and flexural strengths of FC with varying NS by weight fractions. All FC mixtures exhibited a linear relationship, suggesting that the flexural strength can be reliably predicted from the compressive strength.

(2) $$Flexural\;strength=0.369\sqrt{Compressive\;strength}$$

The high R^2^ value (R^2^ = 0.9972) further reinforces the strong linear relationship between these two mechanical properties (flexural and compressive strengths), as expressed in equation (2). This finding highlights the potential of using the compressive strength as an indicator of the flexural strength of FC. With its fine shape, NS provides excellent crack advancement inhibition properties because it blocks and deflects microcracks, giving it powerful properties to inhibit crack advancement under bending and compressive loads. In addition, the bridging ability of NS particles contributes to the arrest and delay of fracture spread to a great extent. Crack coalescence occurs more frequently under tensile strain than under compressive pressure [109]. To verify the proposed model, the compressive strength and flexural strength from the literature [105,106,109] were collected. Fig. 21b demonstrates a comparison between the predicted and actual flexural strengths of FC. While most of the predicted flexural strength values align with the actual flexural strength values, the predictions by Roslan et al. [110] consistently overestimated the actual values. For the remaining predictions, a linear relationship between the predicted and actual flexural strength is evident, indicating the overall reliability of the proposed predictive models.

#### 3.12.3 Correlation between compressive strength and water absorption

**Fig. 22:**
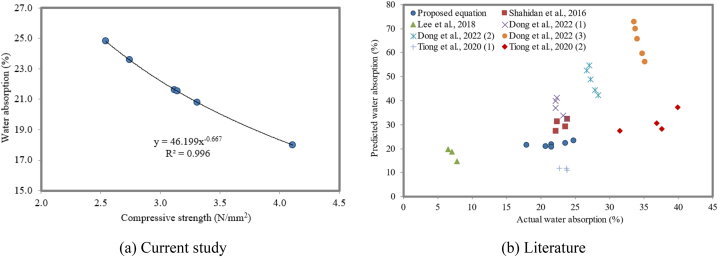
Relationship between compressive strength and water absorption.

The mechanical and permeability properties of concrete are significantly influenced by the porosity of concrete. Hence, it is necessary to correlate the water absorption and the compressive strength of concrete. The proposed relationship between the compressive strength and water absorption is presented in equation (3). The compressive strength and water absorption of FC have a strong negative relationship (R^2^ = 0.996), as depicted in Fig. 22a, and this is true, no matter how much NS is used. This observation aligns with the findings of Ouni et al. [111], who also reported an inverse relationship between these two properties. The higher compressive strength of concrete decreases the porosity, as stated by Hatungimana et al. [112]. This inverse relationship suggests that as the water absorption of FC increases, its compressive strength decreases. This phenomenon is attributed to the presence of pores and voids in the FC microstructure, which weaken the overall structure. An increase in the density of FC results in a reduction of the pores, which leads to an increase in the compressive strength with a decrease in the water absorption.

(3) $$Water\;absorption=46.199{\left(Compressive\;strength\right)}^{-0.667}$$

In order to validate the equation proposed, the compressive strength and water absorption were collected from the literature [[113], [114], [115], [116]]. Fig. 22b reveals that the predicted water absorption values for the third mix by Dong et al. [115] and the second mix by Tiong et al. [116] overestimate the actual water absorption values. This suggests that the predictive models used by these researchers may need to be refined. Higher water absorption in FC is associated with increased pore size and connectivity, which consequently reduces the compressive strength of FC. This highlights the importance of controlling the pore structure to achieve optimal compressive strength in FC.

#### 3.12.4 Correlation between compressive strength and porosity

**Fig. 23:**
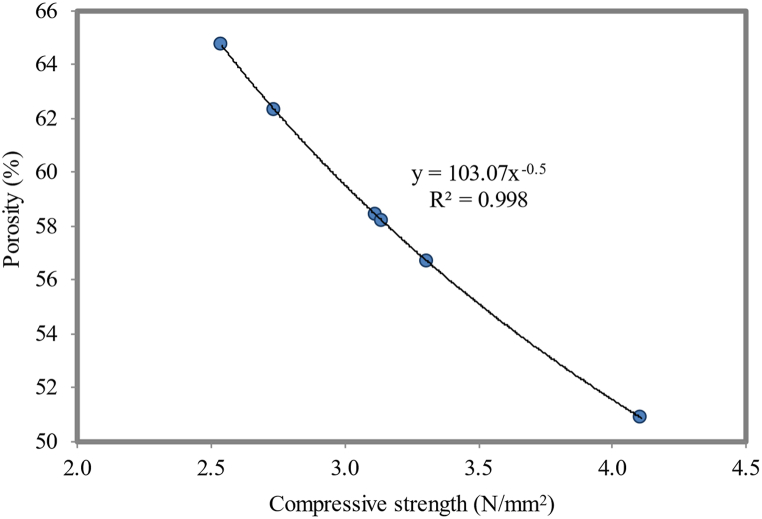
Relationship between compressive strength and porosity.

The inverse relationship illustrates the compressive strength and porosity of FC. An increase in the porosity results in poor bonding between the aggregates and paste, leading to a decrease in the compressive strength. Also, the connectivity of pores decreases the compressive strength. Fig. 23 displays a strong inverse relationship (R^2^ = 0.998) between the compressive strength and porosity of FC. The correlation between the compressive strength and porous concrete within acceptable limits was proposed by Lian et al. [117]. This implies that as the compressive strength of FC increases, its porosity decreases. This observation highlights the inherent connection between these two fundamental properties of FC. The reduction in the porosity with increasing the compressive strength suggests that a denser and more compact microstructure formed, leading to enhanced load-bearing capacity. For NS-blended FC, the relationship between the compressive strength and porosity is shown in equation (4).

(4) $$Porosity=103.07{\left(Compressive\;strength\right)}^{-0.5}$$

#### 3.12.5 Correlation between porosity and water absorption

**Fig. 24:**
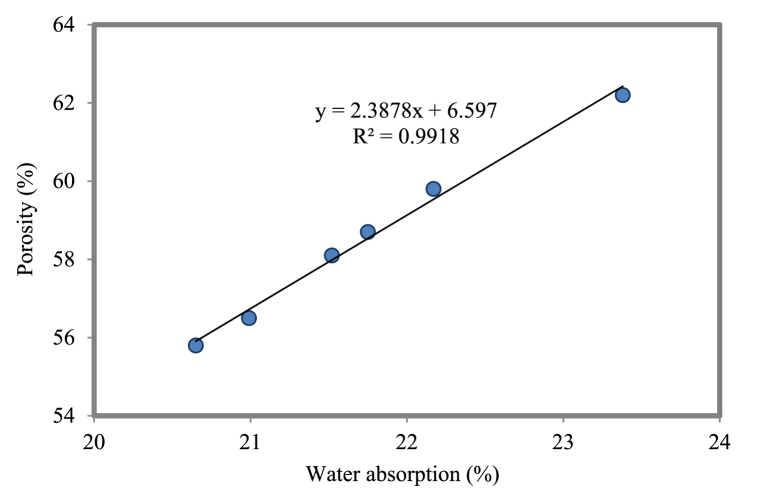
Relationship between porosity and water absorption.

Porosity and water absorption are correlated in Fig. 24 for FC mixtures containing various weight fractions of NS. An R^2^ value of 0.9918 was attained. The connection explains that the water absorption value increases with increasing porosity. NS can inhibit both water absorption and cementitious material capillary absorption. Since NS can be effectively disseminated, a modest dose of NS has the greatest effect on reducing the permeability. Further, it has been demonstrated that the permeability of cementitious materials varies with the NS grain size. This may be due to the fact that NP enhances the microstructure of concrete. Cementitious composites made of NS and FC have more uniform microstructures and less porosity because of the interaction between nanofiller and pozzolanic reactivity, especially at ITZ. This makes FC less porous and less able to absorb water [118]. It is also possible that hazardous substance pathways through the FC cementitious matrix will be partially blocked by the partial filling of this matrix.

## 4 Conclusions

It was found that FC has lower strength and durability properties due to the large number of pores in it. This study explores the prospective utilization of NS as an additive to FC to improve the freshness, strength, and durability properties. A range of the NS weight fractions between 1% and 5% was used in FC with a density of 880 kg/m^3^, and the results were compared to those obtained in a control FC mix. This study can further extend to the other durability properties, and it can even be extended to the application of FC in structural elements. In general, this study indicated that the addition of NS to FC is a substantial factor in developing the properties of FC in terms of the durability and mechanical properties. The following conclusions can be derived from this investigation.

• With an increase in the NS dosages of 1%, 2%, 3%, 4%, and 5%, there was a decrease in the slump of 0.78%, 2.72%, 4.28%, 7.78%, and 8.95%, respectively. The control specimen was considered for FC. With an increase in the NS dosages of 1%, 2%, 3%, 4%, and 5%, there was an increase in the density of about 0.34%, 0.79%, 1.24%, 1.58%, and 2.15%, respectively, compared to the control specimen, which were noted for 28 days of curing.
• When there was an increase in the NS dosages of 1%, 2%, 3%, 4%, and 5%, there was an enhancement in the compressive strength of about 7.81%, 22.78%, 61.81%, 30.31%, and 23.62%, respectively. The control specimen was noted for 28 days of curing. An increase in the NS dosages of 1%, 2%, 3%, 4%, and 5%, improved the splitting tensile strength of about 12.50%, 29.81%, 75.00%, 37.50%, and 28.13%, respectively. The control specimen was noted for 28 days of curing.
• There was an increase in the NS dosages of 1%, 2%, 3%, 4%, and 5%, and an increase in the flexural strength of 7.46%, 22.39%, 58.75%, 30.26%, and 23.47%, respectively. The control specimen was noted for 28 days of curing.
• The increase in the amount of NS from 1% to 5% resulted in a considerable improvement in the porosity, water absorption, intrinsic air permeability, and chloride diffusion properties of FC. In cement matrix-filler interfaces, NS decreased the transport properties of FC by increasing the density of ITZ because of its high reactivity.
• FC that incorporated NS revealed significantly higher thermal conductivity values in comparison to the control FC mix. The main cause of this phenomenon was recognized as the decreased pore size observed in the FC specimens containing NS in relation to the control FC specimen. Consequently, there was an important rise in the thermal insulation of the heat conduction.
• An ideal weight fraction for NS to be added to FC is 3%. By increasing the density of hydration products and reducing the size and interconnectivity of pores, NS increases the mechanical properties of FC. The particles in FC-NS composites are not spread out evenly, which causes particles to stick together and clump, which lowers the mechanical properties. From this study, there is evidence that nanomaterials have the potential to provide a sustainable alternative to non-renewable resources.

## CRediT authorship contribution statement

**Md Azree Othuman Mydin:** Conceptualization, Investigation, Methodology, Project administration, Resources, Software, Supervision, Validation, Formal analysis, Visualization, Writing – original draft, Writing – review and editing. **P. Jagadesh:** Software, Validation, Writing – original draft, Writing – review and editing. **Alireza Bahrami:** Conceptualization, Investigation, Methodology, Project administration, Resources, Software, Supervision, Validation, Formal analysis, Visualization, Writing – original draft, Writing – review and editing. **Anmar Dulaimi:** Project administration. **Yasin Onuralp Özkılıç:** Methodology, Visualization. **Roshartini Omar:** Conceptualization, Investigation, Methodology, Project administration, Resources.

## Declaration of competing interest

The authors declare that they have no known competing financial interests or personal relationships that could have appeared to influence the work reported in this article.
